# Spinal radiographic progression over 2 years in ankylosing spondylitis patients treated with secukinumab: a historical cohort comparison

**DOI:** 10.1186/s13075-019-1911-1

**Published:** 2019-06-07

**Authors:** J. Braun, H. Haibel, M. de Hooge, R. Landewé, M. Rudwaleit, T. Fox, A. Readie, H. B. Richards, B. Porter, R. Martin, D. Poddubnyy, J. Sieper, D. van der Heijde

**Affiliations:** 1 Rheumazentrum Herne, Herne, Germany; 2 Charité Universitätsmedizin Berlin, Berlin, Germany; 3 VIB Inflammation Research Center, Ghent University, Ghent, Belgium; 4 Maastricht University Medical Center, Maastricht, Netherlands; 5 Klinikum Bielefeld, Bielefeld, Germany; 6 Novartis Pharma AG, Basel, Switzerland; 7 Novartis Pharmaceuticals Corporation, East Hanover, USA

**Keywords:** Ankylosing spondylitis, Radiographic progression, Interleukin-17A, Secukinumab, Retrospective cohort study, Biologic therapy, Nonsteroidal anti-inflammatory drugs

## Abstract

**Objective:**

The aim of this study was to compare radiographic progression in patients with ankylosing spondylitis (AS) treated for up to 2 years with secukinumab (MEASURE 1) with a historical cohort of biologic-naïve patients treated with NSAIDs (ENRADAS).

**Methods:**

Baseline and 2-year lateral cervical and lumbar spine radiographs were independently evaluated using mSASSS by two readers, who were blinded to the chronology and cohort of the radiographs. The primary endpoint was the proportion of patients with no radiographic progression (mSASSS change ≤ 0 from baseline to year 2). The Primary Analysis Set included patients with baseline (≤ day 30) and post-baseline day 31–743 radiographs. Sensitivity analyses were performed to assess the robustness of the comparison between the two cohorts, as follows: Sensitivity Analysis Set 1 included all patients with baseline (≤ day 30) and year 2 (days 640–819) radiographs; Sensitivity Analysis Set 2 included all patients with baseline and post-baseline (> day 30) radiographs.

**Results:**

A total of 168 patients (84%) from the MEASURE 1 cohort and 69 (57%) from the ENRADAS cohort qualified for the Primary Analysis Set. Over 2 years, the LS (SE) mean change from baseline in mSASSS for the primary analysis was 0.55 (0.139) for MEASURE 1 vs 0.89 (0.216) for ENRADAS (*p* = 0.1852). Mean changes from baseline in mSASSS were lower in MEASURE 1 vs ENRADAS for the primary and sensitivity analyses. The proportion of patients with no radiographic progression was consistently higher in the MEASURE 1 vs ENRADAS cohort across all cutoffs for no radiographic progression (change in mSASSS from baseline to year 2 of ≤ 0, ≤ 0.5, ≤ 1, and ≤ 2), but the differences were not statistically significant.

**Conclusion:**

Secukinumab-treated patients demonstrated a numerical, but statistically non-significant, higher proportion of non-progressors and lower change in mSASSS over 2 years versus a cohort of biologic-naïve patients treated with NSAIDs.

**Electronic supplementary material:**

The online version of this article (10.1186/s13075-019-1911-1) contains supplementary material, which is available to authorized users.

## Introduction

Ankylosing spondylitis (AS) is a chronic inflammatory disease characterized by inflammation of the sacroiliac joints and the spine that is eventually followed by erosions and new bone formation. Irreversible structural damage occurring as a consequence of new bone formation has a negative impact on patients’ spinal mobility and physical function and may adversely impact their quality of life [1, 2]. Thus, next to the reduction of disease activity, reducing structural damage progression is an important goal in the treatment of patients with AS. Drugs that are effective in both abrogation of spinal inflammation and protection from radiographic damage may have a more beneficial impact on physical function in the long term than drugs that are only effective on one of these domains.

Nonsteroidal anti-inflammatory drugs (NSAIDs) and anti-tumor necrosis factor (TNF) agents have both been demonstrated to improve the signs and symptoms of AS. Although there is some evidence for a potential benefit of NSAIDs in decelerating radiographic progression in AS, when administered continuously [3, 4], there are no data published to date from prospective, randomized, and controlled studies with anti-TNF treatments demonstrating inhibition of spinal radiographic progression in AS [5]. As it is considered unethical to expose patients with AS to placebo treatment for a 2-year period (reported to be the minimum follow-up to detect radiographic progression in an acceptable number of patients), historically controlled NSAID-treated cohort comparisons have been used [6]. Comparison of anti-TNF agents with historical cohorts of biologic-naïve patients treated with NSAIDs has not shown a significant added benefit in reducing radiographic progression at 2 years [7–9].

Interleukin (IL)-17A is a key therapeutic target for the treatment of AS [10]. Secukinumab, a fully human monoclonal antibody that directly inhibits IL-17A [11], was shown to significantly improve the signs and symptoms of AS in patients in the MEASURE 1 core trial (NCT01358175) at 2 years and through 4 years in the extension study (NCT01863732) [12]. A low radiographic progression rate was also reported from the MEASURE 1 core trial through 2 years, and this low rate was sustained through 4 years [12, 13].

This retrospective analysis compared radiographic progression in the spine of patients with AS treated for up to 2 years with secukinumab (MEASURE 1 cohort) with a control cohort of biologic-naïve AS patients (i.e., the effects of nonsteroidal anti-inflammatory drugs [NSAIDs] on Radiographic Damage in Ankylosing Spondylitis [ENRADAS; NCT00715091]) to determine if there were differences in the inhibition of structural damage progression with the two treatments [12, 14].

## Materials and methods

### Patients and study design

This was a retrospective comparative cohort study, at the center of which was an imaging analysis to compare the progression of structural damage in the spine of patients with AS treated with secukinumab for up to 2 years versus patients who had not received biologic therapy. The study compared existing (historical) radiographic data for patients with AS from two cohorts of patients. Patients were treated in 65 centers in 14 countries in the MEASURE 1 study and in 19 centers in Germany in the ENRADAS study.

The MEASURE 1 core study was a randomized phase III trial that enrolled patients ≥ 18 years with AS fulfilling the modified New York Criteria, and active disease as indicated by a Bath Ankylosing Spondylitis Disease Activity Index (BASDAI) score ≥ 4 [15] and a spinal pain score ≥ 4 cm (on a 0–10 cm scale) despite prior treatment with NSAIDs [16]. Eligible patients were anti-TNF naïve or had experienced an inadequate response to anti-TNF or stopped treatment for safety or tolerability reasons (i.e., anti-TNF inadequate responders [anti-TNF IR]) [16]. Patients were randomized to receive a 10 mg/kg intravenous (IV) loading dose at baseline, weeks 2 and 4, and then subcutaneous (SC) injections of 150 (IV→150 mg) or 75 mg (IV→75 mg) every 4 weeks (q4wk) from week 8 [16]. In the placebo-randomized patients, the same IV-to-SC schedule was administered up to week 16 (non-responders) or week 24 (responders), when patients were switched to secukinumab, as previously described [16].

The ENRADAS study was a randomized trial that enrolled patients aged 18–65 years fulfilling the modified New York Criteria and active disease (back pain on a 0–10 numerical rating scale ≥ 4) that justified the start or continuation of an NSAID and had no contraindications for NSAID therapy [14]. Treatment with anti-TNFs was not permitted before or during the study [14]. Patients were randomized to treatment with diclofenac either continuously (at least 50% per day of the maximally recommended daily dose of 150 mg diclofenac, i.e., two pills of 75 mg) or on demand for a total period of 2 years, without a washout period for previous NSAID treatment. Switching to another NSAID was allowed in case of intolerance or inefficacy; equivalent dosages of NSAIDs were used in switchers.

### Radiographic assessments and endpoints

Lateral cervical and lumbar spine radiographs from both cohorts were combined and independently re-evaluated using the mSASSS (range 0–72) scored by two trained readers who were blinded to the chronology and cohort of the radiographs. Radiographic progression was based on the average change from baseline in mSASSS of the two assessors over 2 years. A third trained reader served as an independent adjudicator, who evaluated the top 10% of cases with the highest difference in change in total mSASSS scores between the two primary readers. In the top 10% of cases requiring adjudication, the reading of the third reader was used, with the average readings of the primary readers being used for the remaining 90% of cases.

The primary endpoint was the proportion of patients with no radiographic progression (mSASSS change from baseline to year 2 of ≤ 0) in the MEASURE 1 vs the ENRADAS cohort. Secondary endpoints included a change from baseline in mSASSS to year 2 and the proportion of patients with mSASSS change from baseline to year 2 of ≤ 0.5, ≤ 1, and ≤ 2.

For MEASURE 1, a radiograph performed up to day 30 was considered as baseline; for ENRADAS, the first radiograph determined the baseline. All other study days (e.g., week 104) were labeled relative to day 1. The Primary Analysis Set included all patients with baseline (target day 1; window up to day 30) and post-baseline up to week 104 (target day 729; window day 31 to day 743) radiographs (MEASURE 1, *N* = 168; ENRADAS, *N* = 69). Post-baseline was defined as the measurement closest to the target date. The post-baseline window of day 31 to day 743 was chosen to align with the analysis visit window used in the MEASURE 1 study. In addition, day 743 was chosen as the upper limit for the post-baseline radiograph as 43.4% (53/122) of patients in ENRADAS had a radiograph window greater than 2 years. These patients were not included in the analysis as there would be an increased likelihood of detecting radiographic progression compared with windows less than 2 years. Patients with post-baseline radiographs closer to the lower limit of the window (i.e., day 31) would also have reduced likelihood of showing radiographic progression.

### Statistical analyses

Demographics and baseline characteristics of the two cohorts were summarized and compared. Progressors and non-progressors as per mSASSS changes from baseline with different definitions for no-progression were evaluated using a logistic regression model, with the cohort as a factor and baseline mSASSS as a covariate. Change in mSASSS from baseline to 2 years was calculated as least-squares (LS) mean (standard error [SE]) using an analysis of covariance (ANCOVA) model, with the cohort as a factor and baseline mSASSS as a covariate. Cumulative probability plots were generated for the change in mSASSS values from baseline.

During the conduct of the study, it became apparent that there were differences in the timing of the post-baseline X-ray in the MEASURE 1 and ENRADAS cohorts, which meant that some patients were treated for less than or more than 2 years at the time of their post-baseline imaging assessment. To address this issue, sensitivity analyses were performed to assess the robustness of the comparison between the two cohorts using the following sets: Sensitivity Analysis Set 1 included all patients with baseline (≤ day 30) and year 2 ± 3 months (days 640–819) radiographs; Sensitivity Analysis Set 2 included all patients with baseline (≤ day 30) and post-baseline (> day 30) radiographs (paired radiographs were analyzed adjusted for the difference in time between baseline and post-baseline radiographs as an additional covariate in the model).

An exploratory analysis was added to evaluate inter-reader reliability using the intra-class correlation coefficient (ICC) for mean change from baseline in mSASSS. The smallest detectable change (SDC) for mean change from baseline in mSASSS was calculated based on the 95% level of agreement between the two readers.

## Results

### Patients and radiograph measurements

A total of 168 patients (84%) from the MEASURE 1 cohort and 69 patients (57%) from the ENRADAS cohort qualified for the Primary Analysis Set; 175 (87%) patients from the MEASURE 1 cohort and 78 (64%) patients from the ENRADAS cohort qualified for Sensitivity Analysis Set 1. Mean age, gender, and mSASSS were comparable across cohorts and analysis sets (Table 1) At baseline, patients had higher mean C-reactive protein (CRP) levels (18.3 vs 8.8), and a lower prevalence of smoking (25.0% vs 44.9%) in the MEASURE 1 vs ENRADAS cohorts, respectively. A higher rate of corticosteroid use was observed in ENRADAS (63.9%) vs MEASURE 1 (12.9%) in the full analysis set (i.e., Sensitivity Analysis Set 2).

**Table 1:** Demographics and baseline characteristics of patients in the MEASURE 1 and ENRADAS cohorts

		MEASURE 1			ENRADAS	
Characteristic	Primary Analysis Set (*N* = 168)^a^	Sensitivity Analysis Set 1 (*N* = 175)^b^	Sensitivity Analysis Set 2 (*N* = 201)	Primary Analysis Set (*N* = 69)^a^	Sensitivity Analysis Set 1 (*N* = 78)^b^	Sensitivity Analysis Set 2 (*N* = 122)
mSASSS	9.6 ± 14.1	9.0 ± 14.05	6.4 ± 10.4	9.9 ± 13.8	9.8 ± 13.3	7.9 ± 10.5
CRP (mg/L)	18.3 ± 23.4	18.1 ± 22.1	17.5 ± 21.1	8.8 ± 9.7	10.1 ± 11.5	10.1 ± 12.0
Smoker, *n* (%)	42 (25.0)	33 (18.9)	45 (22.4)	31 (44.9)	35 (44.9)	56 (46.3)
Time since diagnosis, years	7.2 ± 8.0	6.3 ± 7.0	6.9 ± 7.7	10.3 ± 11.1	8.9 ± 10.5	8.6 ± 10.1
NSAID use, *n* (%)	166 (98.8)	174 (99.4)	199 (99.0)	69 (100)	78 (100)	122 (100)
Corticosteroid use, *n* (%)	23 (13.7)	23 (13.1)	26 (12.9)	Not available	Not available	78 (63.9)^c^
Age in years	41.0 ± 12.6	40.3 ± 12.8	41.3 ± 12.6	42.6 ± 10.8	42.0 ± 10.4	42.8 ± 10.2
Male, *n* (%)	123 (73.2)	122 (69.7)	141 (70.1)	46 (66.7)	56 (71.8)	84 (68.9)
HLA-B27 positive, *n* (%)	136 (82.9)	139 (81.8)	155 (79.5)	61 (88.4)	72 (92.3)	110 (90.2)

Data are mean ± standard deviation unless otherwise stated

*CRP* C-reactive protein, *ENRADAS* effects of nonsteroidal anti-inflammatory drugs (NSAIDs) on Radiographic Damage in Ankylosing Spondylitis, *HLA* human leukocyte antigen, *mSASSS* modified Stoke Ankylosing Spondylitis Spinal Score, *NSAID* nonsteroidal anti-inflammatory drugs

^a^Data are shown for patients with baseline and days 31–743 radiographs

^b^Data are shown for patients with baseline and days 640–819 radiographs

^c^Corticosteroid use from the total ENRADAS study population—personal communication from D Poddubnyy

**Fig. 1:**
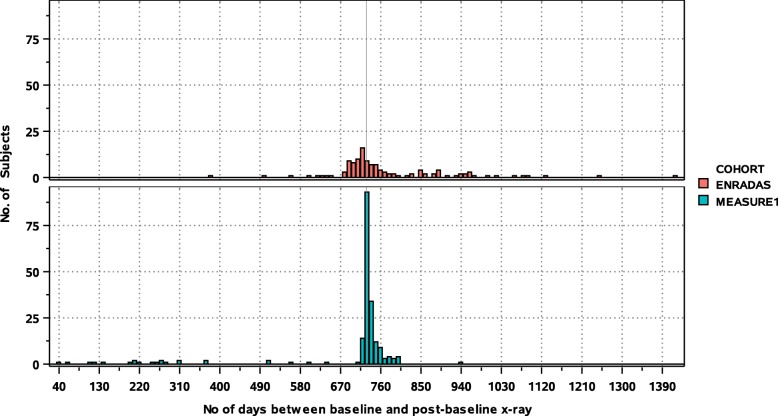
Distributions of the timings of post-baseline radiograph measurements for MEASURE 1 and ENRADAS

Distributions of the timing of post-baseline radiographs for the full analysis sets of MEASURE 1 (*N* = 201) and ENRADAS (*N* = 122) are shown in Fig. 1. The mean (SD) number of days between baseline and post-baseline radiographs for the MEASURE 1 and ENRADAS cohorts were 659.6 (170.8) and 695.1 (56.1) days for the Primary Analysis Set, 737.0 (18.3) and 723.5 (29.3) days for Sensitivity Analysis Set 1, and 688.0 (153.4) and 775.9 (135.6) days for Sensitivity Analysis Set 2.

### Change in mSASSS from baseline

#### Primary analysis

**Fig. 2:**
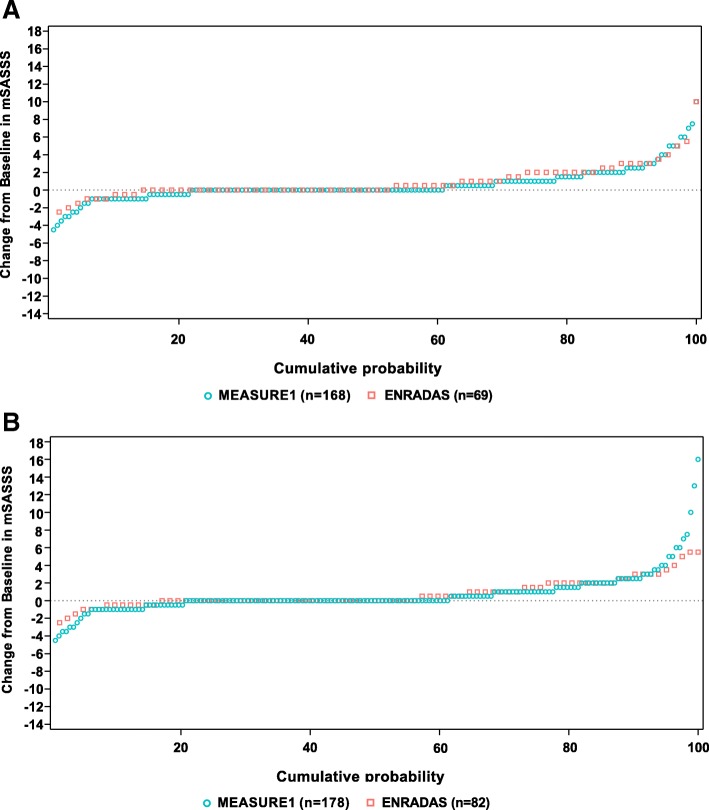
Cumulative probability plot for the change from baseline to year 2 in MEASURE 1 and ENRADAS cohorts using **a** Primary Analysis Set^a^, **b** Sensitivity Analysis Set 1^b^. ^a^Includes patients with baseline (≤ day 30) and days 31–743 radiographs; ^b^Includes patients with baseline (≤ day 30) and day 640–819 radiographs. mSASSS, modified Stoke Ankylosing Spondylitis Spinal Score

The cumulative probability plot illustrates the change in mSASSS from baseline to 2 years for MEASURE 1 and ENRADAS patients in the Primary Analysis Set (Fig. 2a). The proportion of patients with no radiographic progression (LS mean change in mSASSS from baseline to year 2 ≤ 0) was 60.7% in the MEASURE 1 cohort vs 52.2% in the ENRADAS cohort (*p* = 0.2430; Table 2).

**Table 2:** Proportion of patients with no radiographic progression according to mSASSS cut-offs of ≤0, ≤0.5, ≤1.0, and ≤ 2.0

	MEASURE 1 (*N* = 201); ENRADAS (*N* = 122) n (% of N)	No radiographic progression, % of patients			
		∆ ≤0	∆ ≤0.5	∆ ≤1.0	∆ ≤2.0
Primary analysis set (BL to Days 31–743)	M: 168 (84%); E: 69 (57%)	M: 60.7%; E: 52.2%	M: 68.5%; E: 62.3%	M: 78.0%; E: 69.6%	M: 82.1%; E: 72.5%
Sensitivity analysis set 1 (BL to Days 640–819)	M: 178 (89%); E: 82 (67%)	M: 61.2%; E: 56.1%	M: 68.0%; E: 63.4%	M: 77.5% E: 72.0%	M: 81.5%; E: 75.6%
Sensitivity analysis set 2 (All patients, time-adjusted)^a^	M: 201 (100%); E: 122 (100%)	M: 59.8%; E: 50.1%	M: 68.5%; E: 62.4%	M: 79.2%; E: 72.1%	M: 84.6%; E: 75.4%

*BL* Baseline, *E* ENRADAS, *M* MEASURE 1, *mSASSS* Modified stoke ankylosing spondylitis spinal score, *n* Number of patients per cohort in each analysis set, *N* Total number of patients per cohort

^a^All patients with BL (≤ Day 30) and post-BL (> Day 30) radiographs, adjusted for difference in time between BL and post-BL radiographs

#### Secondary analyses

The proportion of patients with no radiographic progression was consistently higher in the MEASURE 1 vs ENRADAS across all cutoffs for no radiographic progression (change in mSASSS from baseline to year 2 of ≤ 0, ≤ 0.5, ≤ 1 and ≤ 2; Table 2), although the results were not statistically significant. Over 2 years, the LS (SE) mean change from baseline in mSASSS for the Primary Analysis Set was 0.55 (0.139) for MEASURE 1 vs 0.89 (0.216) for ENRADAS (*p* = 0.1852; Table 3).

**Table 3:** LS mean change from Baseline in mSASSS and the different in LS means in MEASURE 1 vs ENRADAS

	MEASURE 1 (*N* = 201); ENRADAS (*N* = 122) n (% of N)	LS mean change (SE) from BL in mSASSS	Difference in LS means (SE) (MEASURE 1 vs. ENRADAS) *p* value
Primary analysis set (BL to Days 31–743)	M: 168 (84%); E: 69 (57%)	M: 0.55 (0.139); E: 0.89 (0.216)	−0.34 (0.257); *p* = 0.1852
Sensitivity analysis set 1 (BL to Days 640–819)	M: 178 (89%); E: 82 (67%)	M: 0.69 (0.153); E: 0.72 (0.225)	−0.03 (0.272); *p* = 0.9175
Sensitivity analysis set 2, (All patients)^a^	M: 201 (100%); E: 122 (100%)	M: 0.68 (0.168); E: 0.99 (0.217)	−0.31 (0.280); *p* = 0.2636

*BL* Baseline, *E* ENRADAS, *LS* Least squares, *M* MEASURE 1, *mSASSS* Modified stoke ankylosing spondylitis spinal score, *SE* Standard error, *n* Number of patients per cohort in each analysis set, *N* Total number of patients per cohort

^a^All patients with BL (≤ Day 30) and post-BL (> Day 30) radiographs, adjusted for the difference in time between BL and post-BL radiographs

Cumulative probability plots illustrating the change in mSASSS from baseline using Sensitivity Analysis Set 1 is shown in Figs. 2b. Differences in the LS mean change from baseline in mSASSS were lower in the MEASURE 1 vs ENRADAS cohorts across all of the sets analyzed (Table 3).

#### Reader reliability

The ICC for the mean change from baseline in mSASSS was 0.17 in the Primary Analysis Set. The SDC with a 95% level of agreement was 3.7 for the Primary Analysis Set.

## Discussion

The progression rate of patients treated with secukinumab found in this study was in the same range as in the recent publications [12–14]. Evaluating the Primary Analysis Set of this study, we found a numerically higher proportion of radiographic non-progressors (defined as an mSASSS change from baseline ≤ 0 at year 2 [primary outcome]) among secukinumab-treated patients (MEASURE 1) compared with controls from a cohort of biologic-naïve NSAID-treated patients (ENRADAS) over 2 years. However, differences between these groups were not statistically significant. The proportion of non-progressors was consistently higher in the MEASURE 1 vs ENRADAS cohorts across all sets analyzed and regardless of the mSASSS cutoff used (mSASSS change from baseline ≤ 0, ≤ 0.5, ≤ 1 or ≤ 2 at year 2). In this analysis, around 82–85% of patients in MEASURE 1 exhibited inhibition of radiographic progression using an mSASSS cutoff of ≤ 2 at year 2 across all sets analyzed compared with around 71–76% for ENRADAS.

It is possible that the differences in time intervals between baseline and 2-year radiographs in MEASURE 1 vs ENRADAS may have confounded the results in the Primary Analysis Set owing to the wide window for post-baseline radiographs. To address this limitation, Sensitivity Analysis Set 1 was included with a narrow window for the post-baseline radiograph around 2 years (i.e., days 640–819). The results of this analysis confirmed a higher proportion of radiographic non-progressors in MEASURE 1 (81.5%) vs ENRADAS (75.6%). Results of Sensitivity Analysis Set 2, which was adjusted for the differences in timings of the baseline and post-baseline radiographs, confirmed these findings with an even higher rate of radiographic non-progressors in MEASURE 1 (84.6%) vs. ENRADAS (75.4%). A cutoff of 2 years was chosen in the current study as some patients in ENRADAS had their post-baseline radiographs after 2 years, resulting in a longer radiographic interval compared with MEASURE 1.

Historically controlled cohort comparisons with anti-TNF agents have shown no benefit of anti-TNF therapies compared with NSAIDs on structural progression after 2 years (range of mean change in mSASSS was 0.8–0.9 in patients treated with anti-TNF agents vs 0.9–1.0 NSAID-treated biologic-naïve patients) [7–9]. While prospectively controlled studies with anti-TNF agents have not demonstrated a reduction in radiographic structural progression over 2 years, recent 4-year data with certolizumab treatment demonstrated a low rate of mSASSS progression, with non-progression seen in 80.6% of patients [17]. Longitudinal data from cohort studies have suggested that a longer duration of treatment with anti-TNFs of 4 to 8 years may be required before a positive effect on radiographic structural progression is observed [18–21].

In the MEASURE 1 study, treatment with secukinumab resulted in a low mean change in mSASSS from baseline to 2 years of 0.3 (SD 2.53) overall, and 0.38–0.52 among patients with known predictors of radiographic progression at baseline, such as syndesmophytes or elevated CRP [12]. In the extension study, 79% of patients treated with secukinumab 150 mg reported no radiographic progression (change in mSASSS from baseline < 2) at 4 years [13].

Further research is needed to understand the impact of IL-17A inhibition with secukinumab on spinal disease progression in AS patients. Since no direct comparison between anti-TNF agents and IL-17 blocking agents has been performed so far it can be expected that SURPASS (NCT03259074), an ongoing head-to-head study powered to compare differences in spinal radiographic progression with secukinumab compared with biosimilar adalimumab, will help to address this [22].

Due to the retrospective nature of this study and lack of randomization/stratification of patients between the study cohorts, there was some heterogeneity between the two cohorts (i.e., higher CRP levels, lower corticosteroid use, and a lower proportion of smokers in the MEASURE 1 vs ENRADAS cohorts). This may be viewed as a limitation of the study, as patient and disease characteristics at baseline can increase the risk of structural progression [23–25]. In addition to the timings of the post-baseline radiographs, the differences in time between when the two studies were performed may also have confounded the results, as there may have been differences between the populations owing to changes in the environment, lifestyle, and other unknown factors.

## Conclusions

The key findings of this analysis showed that over 2 years, secukinumab-treated patients demonstrated a numerically higher proportion of radiographic non-progressors and a numerically, but statistically non-significant, lower change in mSASSS compared with biologic-naïve NSAID-treated patients in the Primary Analysis Set. Given the variability in timing between the baseline and post-baseline X-ray assessments in the pre-defined Primary Analysis Set, sensitivity analyses were conducted on another set of patients who were confirmed as having closer to 2 years of treatment prior to the post-baseline imaging assessment. The well-powered SURPASS randomized controlled trial will examine the impact of IL-17A inhibition with secukinumab on spinal disease progression in AS patients and will provide a more robust answer.

## Additional file

Additional file 1: **Tables S1.** List of ethical approval reference numbers for each participating center in MEASURE 1. (DOCX 21 kb)

## Abbreviations

ANCOVA: Analysis of covariance
AS: Ankylosing spondylitis
CRP: C-reactive protein
ICC: Intra-reader correlation coefficient
IL-17: Interleukin-17
IR: Inadequate responder
IV: Intravenous
LS: Least squares
mSASSS: Modified stoke ankylosing spondylitis spinal score
NSAID: Nonsteroidal anti-inflammatory drug
q4wk: Every 4 weeks
SD: Standard deviation
SDC: Smallest detectable change
SE: Standard error
TNF: Tumor necrosis factor
