# Supplementary material for: Spinal radiographic progression over 2 years in ankylosing spondylitis patients treated with secukinumab: a historical cohort comparison
Source: Arthritis Res Ther. 2019 Jun 7;21:142. doi: 10.1186/s13075-019-1911-1 (PMC6555995; doi:10.1186/s13075-019-1911-1)
Supplement: Supplementary file 1 — Tables S1. List of ethical approval reference numbers for each participating center in MEASURE 1. (DOCX 21 kb) [file 13075_2019_1911_MOESM1_ESM.docx]

**Table S1. List of Ethical Approval Reference Numbers for Each Participating Center in MEASURE 1**

| **Centre Number** | **Centre Name** | **City, Country** | **Investigator Name** | **Institutional Review Board Reference Number** |
| --- | --- | --- | --- | --- |
| **MEASURE 1** | | | | |
| 2001 | Universitair Ziekenhuis Gent | Gent, Belgium | Filip Van den Bosch | 2013/1063 |
| 2002 | Cliniques Universitaires Saint-Luc | Bruxelles, Belgium | Adrien Nzeusseu | 2013/1063 |
| 2003 | Gasthuisberg University Hospital | Leuven, Belgium | Kurt de Vlam | 2013/1063 |
| 2004 | Reuma Clinic | GENK, Belgium | Piet Geusens | 2013/1063 |
| 2011 | Military Medical Academy- MHAT-Sofia | Sofia, Bulgaria | Boycho Oparanov | № КИ-262/27.12.2013 |
| 2012 | MHAT Sveti Ivan Rilksi Rheumatology Clinic | Sofia, Bulgaria | Rasho Rashkov | № КИ-262/27.12.2013 |
| 2013 | MHAT Kaspela EOOD | Plovdiv, Bulgaria | Anastas Batalov | № КИ-262/27.12.2013 |
| 2014 | MHAT Plovdiv AD | Plovdiv, Bulgaria | Ivan Goranov | № КИ-262/27.12.2013 |
| 2015 | MHAT Burgas AD | Burgas, Bulgaria | Ivan Kazmin | № КИ-262/27.12.2013 |
| 2021 | Manitoba Clinic | Winnipeg, Canada | Timothy McCarthy | #Pro00011501 |
| 2025 | St. Clare's Mercy Hospital | St. John's, Canada | Proton Rahman | #13.252 |
| 2032 | Hôpital Pellegrin | Bordeaux Cedex, France | Thierry Schaeverbeke | 2013/72 |
| 2033 | Hopital Cochin | PARIS, France | Minh N Guyen | 2013/72 |
| 2034 | CHU Dupuytren | Limoges cedex, France | Pascale Vergne Salle/ Philippe Bertin | 2013/72 |
| 2061 | Az.Osped.Universitaria Senese Ospedale.S.Maria alle Scotte | Siena, Italy | Bruno Frediani | Not applicable |
| 2063 | Az.Osp.Univ.Policlin.V.Emanuele P.O.Vittorio Emanuele II | Catania, Italy | Rosario Foti | Not applicable |
| 2065 | Az.Osp.Univ.Policlinico P.Giaccone Università Studi Palermo | Palermo, Italy | Giovanni Triolo | Not applicable |
| 2069 | P.O.Molinette AO Città della Salute e della Scienza Torino | Torino, Italy | Enrico Fusaro | Not applicable |
| 2072 | Az. Osp. Spedali Civili di Brescia Univ.degli Studi | Brescia, Italy | Franco Franceschini | Not applicable |
| 2081 | Centro Medico del Angel S.C. | Mexicali, Mexico | Beatriz Zazueta | 13 CEI 09 006 049 |
| 2082 | Centro de Investigación de Tratamientos Innovadores | Culiacan, Mexico | Marco Maradiaga | 13 CEI 09 006 049 |
| 2083 | Centro Integral de Reumatologia S.A de C.V. | Guadalajara, Mexico | Hilario Avila | CONBIOÉTICA-14-CEI-005-20170427 |
| 2084 | Hospital Universitario "Dr. Jose Eleuterio Gonzalez" UANL | Monterrey, Mexico | Mario Garza | CONBIOÉTICA-19-CEI-001-20160404 |
| 2091 | Academisch Medisch Centrum | Amsterdam, Netherlands | D.L. Baeten/ Marleen van de Sande | METC 2013_267 |
| 2092 | UMC Utrecht lok. Heidelberglaan | Utrecht, Netherlands | Jacob van Laar / J.W.J. Bijlsma (Hans) | METC 2013_267 |
| 2101 | Medicentro Bio Ciencias-ABK REUMA | Pueblo Libre, Peru | Alfredo Berrocal | Letter 1713-2014 |
| 2103 | Centro de Investigación en Inmuno Reumatología-Hospital de | Surquillo, Peru | Boris Garro | Letter 178-2014 |
| 2104 | Red Asistencial Guillermo Almenara Irigoyen-EsSALUD | La Victoria, Peru | Rocio Gamboa | Letter 291-2014 |
| 2105 | Clinica Anglo Americana | San Isidro, Peru | Oswaldo Castaneda | CIEL_CAA_109/2014 |
| 2106 | Red Asistencial Edgardo Rebagliati Martins-EsSALUD | Jesus Maria, Peru | Felipe Becerra | 00003285 |
| 2111 | Rheumatology Inst. of Russian Academy of Medical Sciences | Moscow, Russia | Marina Stanislav | 26 |
| 2113 | Regional Clinical Hospital | Tula, Russia | Tatyana Salnikova | 51 |
| 2114 | Federal North-West Medical Research Centre | Saint-Petersburg, Russia | Alexey Maslyanskiy | 212 |
| 2117 | Yaroslavl Soloviyev Clinical Hospital | Yaroslavl, Russia | Olga Ershova | 124 |
| 2118 | Ural State Medical University of Roszdrav | Ekaterinburg, Russia | Nadezda Izmozherova | 8 |
| 2119 | Sverdlovsk regional clinical hospital #1 | Ekaterinburg, Russia | Olga Lesnyak | 104 |
| 2121 | Kaohsiung Veterans General Hospital | Kaohsiung, Taiwan | Jui-Cheng Tseng | VGHKS11-CT11-07 |
| 2122 | Chung Shan Medical University Hospital | Taichung, Taiwan | Cheng-Chung Wei | CS11126 |
| 2135 | Gaziantep University Medical Faculty | Gaziantep, Turkey | Bunyamin Kisacik / Orhan Zengin / Savas Gursoy | 23.06.2014/86 |
| 2137 | Dokuz Eylul University Medical Faculty | Balcova / Izmir, Turkey | Fatos Onen | 23.06.2014/86 |
| 2141 | Whipps Cross Hospital | London, United Kingdom | Hasan Tahir | 13/EM/0357 |
| 2142 | Addenbrookes Hospital | Cambridge, United Kingdom | Andrew Ostor / Deepak Jadon | 13/EM/0357 |
| 2144 | New Cross Hospital | Wolverhampton, United Kingdom | Nick Barkham | 13/EM/0357 |
| 2145 | Royal Victoria Infirmary | Newcastle Upon Tyne, United Kingdom | Lesley Kay | 13/EM/0357 |
| 2151 | Rheumazentrum Ruhrgebiet St.-Josefs-Krankenhaus | Herne, Germany | Juergen Braun | 2013-449-f-A |
| 2155 | Praxis für Klinische Studien Dr. med. Georg Dahmen | Hamburg, Germany | Georg Dahmen | 2013-449-f-A |
| 2159 | Universitaetskliniken Koeln | Koeln, Germany | Andrea Rubbert-Roth/ David Kofler | 2013-449-f-A |
| 2173 | Praxis Nüßlein | Nürnberg, Germany | Hubert Nuesslein | 2013-449-f-A |
| 2176 | Institut für Präventive Medizin & Klinische Forschung GbR | Magdeburg, Germany | Ruediger Moericke | 2013-449-f-A |
| 2178 | Universitätsklinikum Erlangen-Nürnberg | Erlangen, Germany | Juergen Rech | 2013-449-f-A |
| 2179 | Charité Berlin, Campus Benjamin Franklin | Berlin, Germany | Joachim Sieper/ Denis Poddubnyy | 2013-449-f-A |
| 5301 | Oregon Health & Science University | Portland, United States | Atul Deodhar | # 28594 |
| 5302 | Altoona Center for Clinical Research | Duncansville, United States | Alan Kivitz | # 28594 |
| 5303 | West Tennessee Research Institute | Jackson, United States | Jacob Aelion | # 28594 |
| 5308 | Arthritis Northwest, PLLC | Spokane, United States | Sean LaSalle | # 28594 |
| 5311 | Holston Medical Group | Kingsport, United States | Song Zang | # 28594 |
